# Progressive ganglion cell loss and optic nerve degeneration in DBA/2J mice is variable and asymmetric

**DOI:** 10.1186/1471-2202-7-66

**Published:** 2006-10-03

**Authors:** Cassandra L Schlamp, Yan Li, Joel A Dietz, Katherine T Janssen, Robert W Nickells

**Affiliations:** 1 Department of Ophthalmology and Visual Sciences, University of Wisconsin, Madison, WI, USA

## Abstract

**Background:**

Glaucoma is a chronic neurodegenerative disease of the retina, characterized by the degeneration of axons in the optic nerve and retinal ganglion cell apoptosis. DBA/2J inbred mice develop chronic hereditary glaucoma and are an important model system to study the molecular mechanisms underlying this disease and novel therapeutic interventions designed to attenuate the loss of retinal ganglion cells. Although the genetics of this disease in these mice are well characterized, the etiology of its progression, particularly with respect to retinal degeneration, is not. We have used two separate labeling techniques, post-mortem DiI labeling of axons and ganglion cell-specific expression of the βGeo reporter gene, to evaluate the time course of optic nerve degeneration and ganglion cell loss, respectively, in aging mice.

**Results:**

Optic nerve degeneration, characterized by axon loss and gliosis is first apparent in mice between 8 and 9 months of age. Degeneration appears to follow a retrograde course with axons dying from their proximal ends toward the globe. Although nerve damage is typically bilateral, the progression of disease is asymmetric between the eyes of individual mice. Some nerves also exhibit focal preservation of tracts of axons generally in the nasal peripheral region. Ganglion cell loss, as a function of the loss of βGeo expression, is evident in some mice between 8 and 10 months of age and is prevalent in the majority of mice older than 10.5 months. Most eyes display a uniform loss of ganglion cells throughout the retina, but many younger mice exhibit focal loss of cells in sectors extending from the optic nerve head to the retinal periphery. Similar to what we observe in the optic nerves, ganglion cell loss is often asymmetric between the eyes of the same animal.

**Conclusion:**

A comparison of the data collected from the two cohorts of mice used for this study suggests that the initial site of damage in this disease is to the axons in the optic nerve, followed by the subsequent death of the ganglion cell soma.

## Background

In a systematic examination of intraocular pressure (IOP) in inbred mice, John and colleagues described elevated levels of IOP and the subsequent development of an optic neuropathy in the DBA/2J (D2) line [1]. The development of elevated IOP in these mice is linked to mutations in two genes, *Gpnmb *and *Tyrp1*, which encode a protein found in melanosomal membranes and an enzyme involved in melanin synthesis, respectively [2,3]. Recessive inheritance of both of these mutant genes causes the breakdown of the iris stroma and the release of pigment clumps into the anterior chamber of the eye. The association of these proteins with melanosomes has lead to the theory that toxic byproducts generated by the biosynthesis of melanin are released from the melanosome leading to the atrophy of the iris. In some respects, this disease resembles human pigment dispersion syndrome in that displaced pigment accumulates in the trabecular meshwork (TM) leading to elevated IOP and glaucoma. It is also clear that disease in the D2 mouse has an immune component that may contribute to both iris atrophy and the pathology of the TM. D2 mice exhibit a decrease in ocular immune privilege as they age. Leakage of the blood brain barrier leads to infiltration of monocytes and neutrophils into the anterior chamber and the iris [4], possibly in response to increasing amounts of toxic melanin byproducts accumulating in the anterior chamber. Bone marrow transplants into D2 mice, from genetically different donors, can effectively prevent the age-related decrease in immune privilege leading to a substantial reduction in both the anterior chamber disease and the subsequent increase in IOP.

The temporal course of the pathology of the anterior chamber disease is relatively predictable, but by no means synchronous in D2 mice. In general, defects in the iris, as determined by transillumination, begin to occur in mice at ~6 months of age. Elevation in IOP is detected in animals anytime from 9–12 months, and is variable within the population [5]. Early studies of the time course of retinal ganglion cell death indicated that a majority of animals exhibited significant cell loss and optic nerve degeneration by 12 months [1] and TUNEL studies showed that peak cell death in the ganglion cell layer of these mice occurred between 10 and 13 months [6]. Similarly, DBA/2NNia mice, a substrain of the DBA/2J line, also exhibited ganglion cell loss between 12 and 15 months of age [7-9]. Several studies have characterized the neuronal populations affected in this disease. Jakobs and colleagues described that the dying cells at this age were almost entirely made up of different sub-types of retinal ganglion cells, while other retinal cell-types, such as amacrine cells, were unaffected [10]. A study by Moon et al showed a similar depletion of ganglion cells, but also noted changes in a subset of amacrines [11]. An ultrastructural electron microscope study, conducted by Scheuttauf and colleagues, described two patterns of neurodegeneration as D2 mice age [12]. Although this study relied on qualitative observations, it concluded that ganglion cell apoptosis was more prevalent in mice under 6 months of age, while necrotic cell death was more prevalent in older mice. In addition to necrosis, mice at ages of 8 and 11 months exhibited ischemic retinal changes, and showed evidence of activated Müller's cells and an increase in neoangiogenesis. Aged retinas showed no signs of inflammatory cells or damage to other retinal cell layers, such as the photoreceptors. These observations may be quite significant, because they indicated that necrosis was the principal mechanism of cell death during the period of elevated IOP. This assessment was made using relatively general morphological criteria on very few animals, however, and more recent studies using *Bax *knock out animals have demonstrated that intrinsic apoptosis is the primary pathway of cell death associated with elevated IOP in D2 mice [6].

In this study, we report on the timing and pattern of both optic nerve and retinal degeneration in two separate cohorts of D2 mice. In both cases, the disease exhibits variable and asymmetric progression in mice as they age and has features that are consistent with glaucoma in humans. Additionally, a comparison of the onset of damage between the two, suggests that optic nerve degeneration precedes measurable damage in the retina, implicating the optic nerve as the initial site of damage in this disease.

## Results

### Evaluation of optic nerve degeneration in aging D2 mice

**Figure 1:**
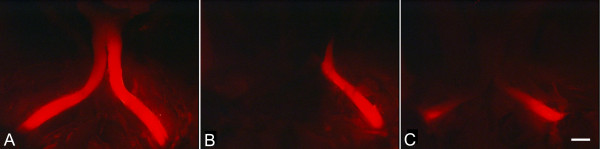
**Photomicrographs of the optic nerves of 3 mice labeled post-mortem with DiI**. The images shown are dorsal views, looking down on the mouse head with the nose of the mouse facing the bottom of the image. The right nerve is on the left of each photomicrograph. (A) A young mouse (6 months of age) showing both optic nerves labeled from the globes to the optic chiasm. (B) An old mouse (10 months of age) showing relatively symmetric degeneration of both nerves. Degenerating nerves typically exhibit reduced label distally. (C) A second mouse at 10 months of age, showing asymmetric degeneration of it optic nerves. Only the left nerve is labeled. Bar = 0.5 mm.

A cohort of 270 nerves from 135 D2 mice, aged between 6 and 22.5 months, was used in this analysis. Mice were euthanized and the nerves labeled with DiI as described in the methods. Figure 1 shows examples of labeling from young (Fig. 1A) and aged mice (Figs. 1B and 1C). Young mice typically showed robust DiI labeling extending to the optic chiasm. Mice that exhibited degeneration of their nerves typically fell into the general categories of those showing symmetric (Fig. 1B) or asymmetric degeneration (Fig. 1C). In this latter group, some mice exhibited one relatively healthy appearing nerve and nearly complete loss of staining in the other. Further evaluation of the asymmetry of degeneration in these mice is presented below.

**Figure 2:**
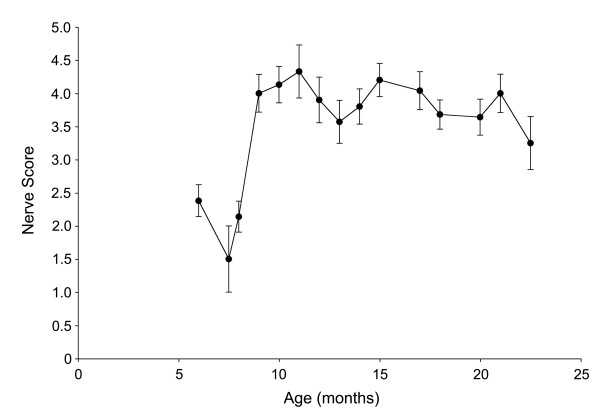
**Graph of the mean (± SEM) severity score for individual optic nerves of mice as a function of age**. This cohort of DBA/2J mice showed a steep increase in the prevalence of optic nerve degeneration at 9 months of age.

**Figure 3:**
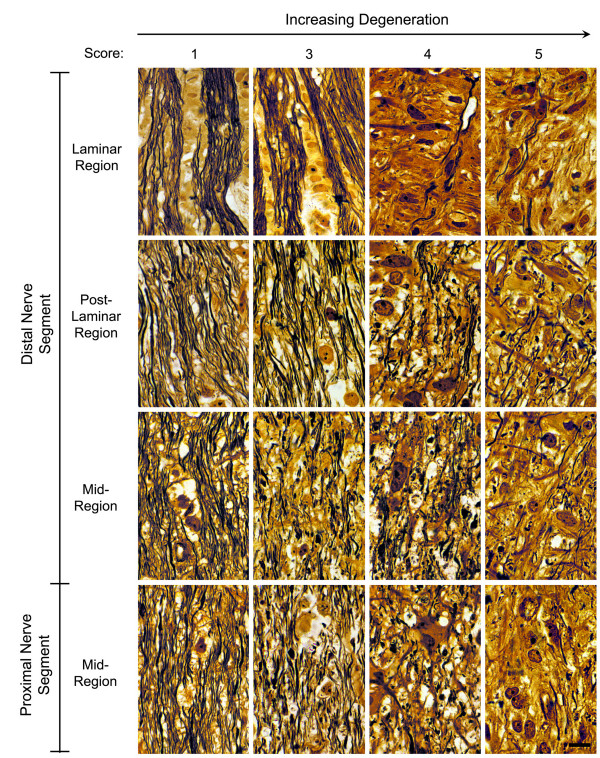
**Degeneration of axons occurs first in regions of the optic nerve proximal to the laminar region**. A series of 16 photomicrographs are shown of 4 different optic nerves in different stages of degeneration based on the DiI-labeling pattern. The distal and proximal segments of each nerve were sectioned longitudinally and silver stained. The individual nerves are oriented from left to right, with their respective score shown at the top. Photomicrographs taken from each nerve are oriented from the laminar region (top panels) through to a region in the middle of the proximal segment (bottom panels). A normal nerve (left panels – score of 1) contains bundles of axons flanking columns of cells in the laminar region. Immediately posterior to this region, the axons separate from the bundles and anastomose along the entire length of the nerve. A nerve with moderate degeneration (panels second from the left – score of 3) also contains relatively normal appearing bundles of axons in the lamina. Small regions of degenerating axons are found along nerve posterior to the lamina, especially in the mid-region of the distal segment and the proximal segment. These regions are exemplified by swollen axons and axon fragments. Nerves with severe degeneration (panels third and fourth from the left) show much more extensive axonal degeneration and loss, gliosis, and scar tissue deposition. Bar = 15 μm.

To quantify the extent of optic nerve degeneration in these mice, the pattern of label in each nerve was scored by 2 masked observers as described in the Methods. The time course of degeneration was then estimated by graphing the mean scores (± SEM) of nerves at each age (Fig. 2). Only a few mice aged 8 months or younger showed signs of degeneration using this labeling method. After 8 months the average level of degeneration rose dramatically and peaked at 11 months of age. The general pattern of age-related degeneration, as indicated by the DiI labeling technique, suggested that axonal degeneration followed a die-back pattern from the proximal end of the optic nerve to the distal end. To assess this pattern, we examined several nerves in different stages of disease histologically in transverse sections. Figure 3 shows a panel of silver stained sections taken at different intervals along 4 nerves. Surprisingly, nerves with early to moderate signs of degeneration using the DiI method (i.e., grades 2 and 3 nerves) showed mostly normal axon tracts throughout most of their length, but contained intermittent regions of degeneration beginning posterior of the lamina. These regions were marked by swollen axons and axonal fragments and could be found throughout the nerve, but were more prevalent in the proximal segment. Regions of degeneration were much more extensive in grade 4 nerves, while grade 5 nerves exhibited fewer axonal fragments and an increase in gliosis and connective tissue deposition.

**Figure 4:**
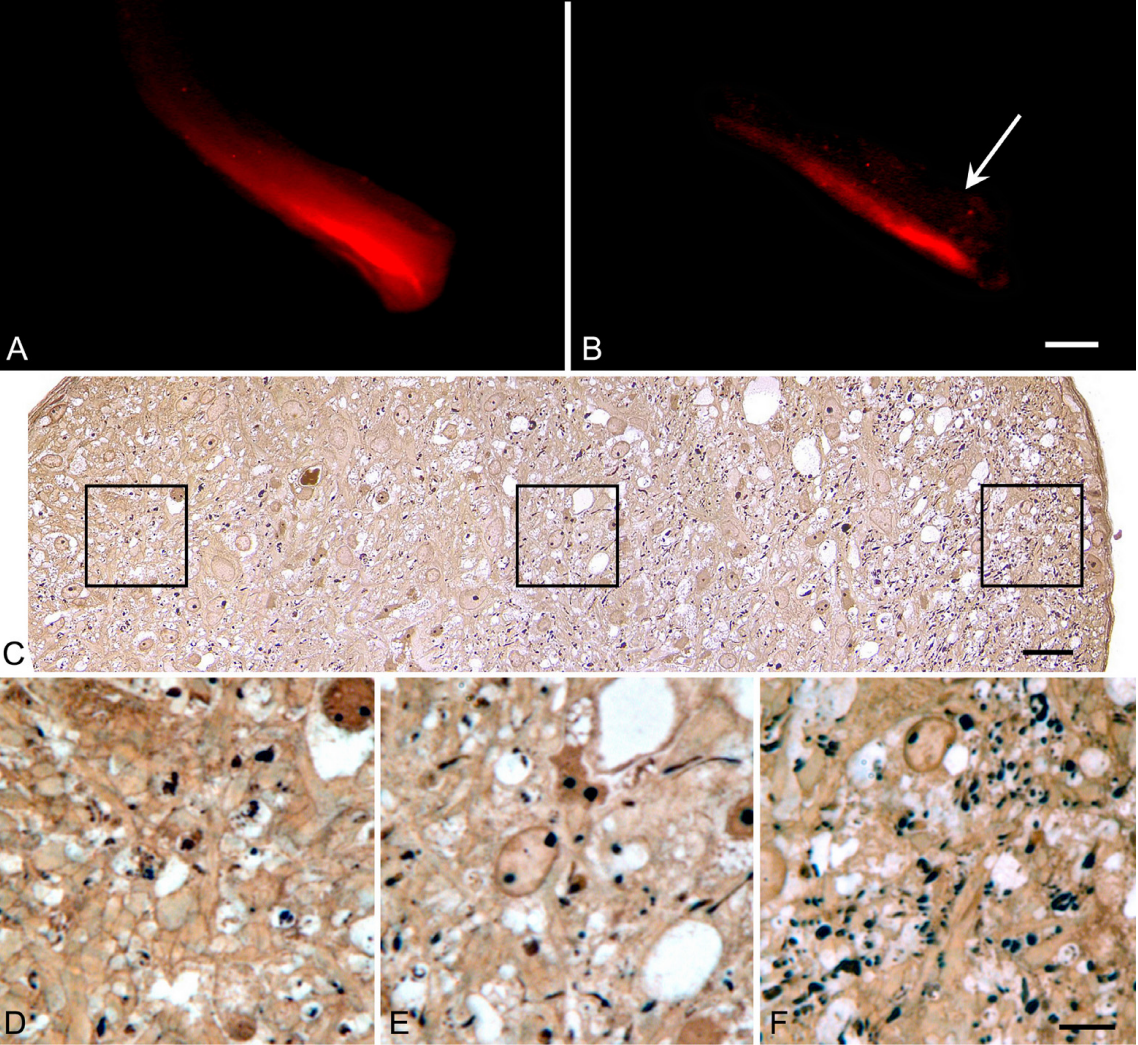
**Nerves with severe degeneration often show preservation of nasal tracts of axons**. (A, B) DiI-labeled optic nerves showing nasal tracts of staining. The left nerve only is shown for each mouse. The nerve in panel B is almost completely degenerated. The edge of the nerve sheath on the temporal side is marked with an arrow. Bar = 0.6 mm. (C) Silver-stained cross section of a nerve showing a peripheral tract of DiI-staining. This low resolution montage was made up of a series of photomicrographs taken at 1000X. The nasal part of the nerve is oriented to the right. Bar = 15 μm. (D-F) Higher resolution images of the respective boxed region in (C) shown above each panel. (D) Temporal optic nerve. (E) Central optic nerve. (F) Nasal optic nerve. The nasal region of this nerve contains a higher density of axons, consistent with the DiI-labeling pattern. Bar in (F) = 4 μm.

The DiI also revealed an interesting pattern of degeneration in approximately 26% of the nerves examined. In these nerves, intact tracts of axons appeared to be preserved at the periphery of the nerve, principally along the nasal side (Fig. 4). Histological evaluation of these nerves confirmed that the pattern of DiI label corresponded to a higher density of axons localized to the nerve periphery.

### Evaluation of retinal ganglion cell loss in aging D2 mice

**Figure 5:**
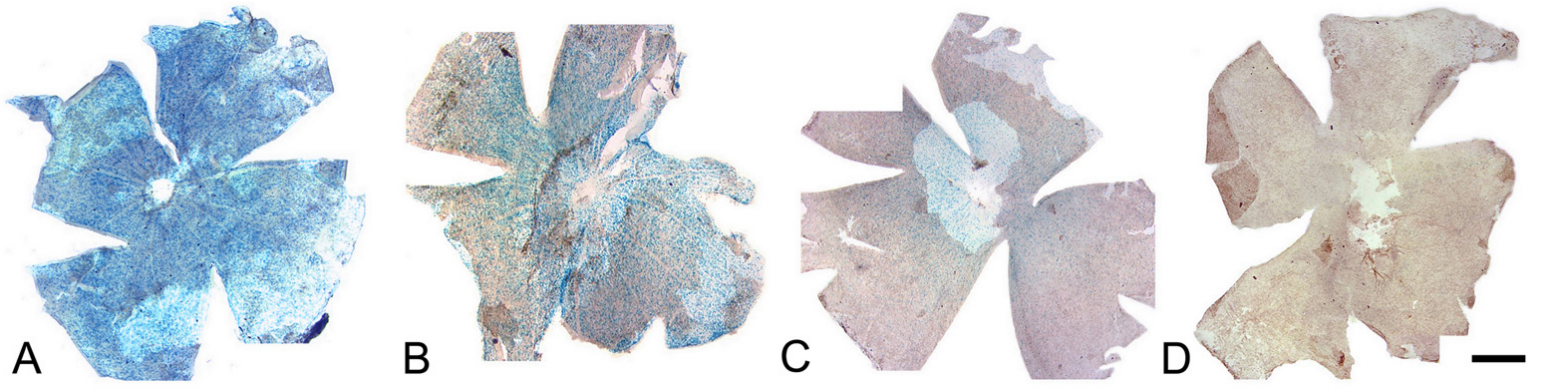
**Whole mounts of retinas taken from 13 month old DBA/2J^R3/+ ^mice show variable levels of degeneration**. Retinas were stained for βGEO activity in the presence of X-Gal. Each retina is taken from a different animal. This series of photomicrographs demonstrates the high degree of variability observed in disease progression in the DBA/2J line, where some animals have virtually no evidence of ganglion cell loss (A), while others have nearly complete cell loss (D). The retinas shown also demonstrate the scoring system used to quantify the βGeo staining patterns observed: (A) represents a score of approximately 1, (B) a score of approximately 2, (C) a score of approximately 3, and (D) a score of approximately 4. Retinas given a score of 5 exhibited no positively staining cells. Bar = 1 mm.

**Figure 6:**
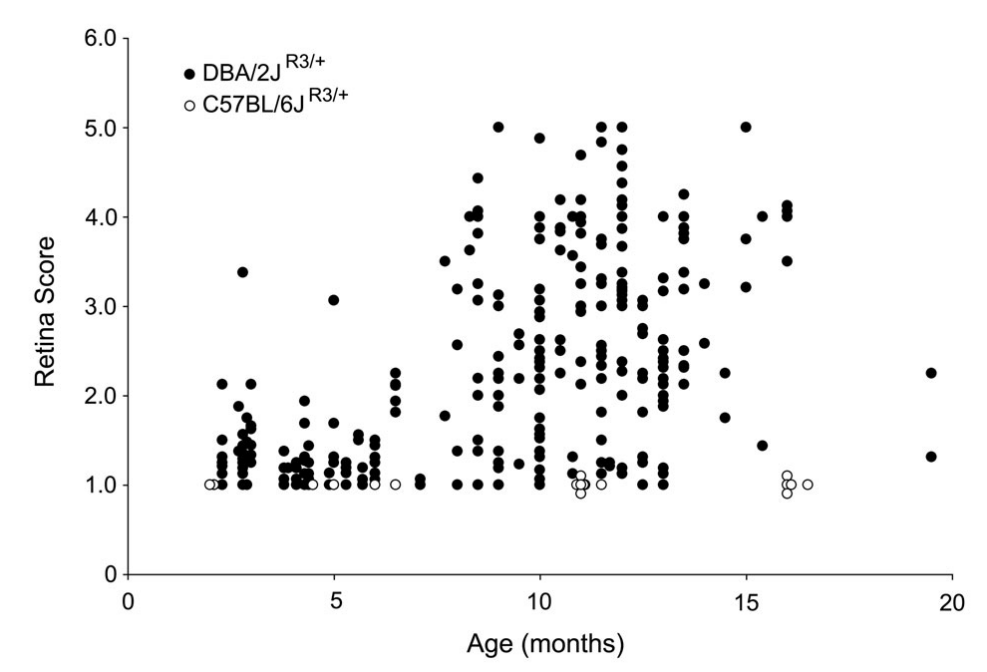
**Scatter plot of X-Gal staining scores for individual retinas from aged DBA/2J ^R3/+ ^mice compared to aged C57BL/6^R3/+ ^animals**. Loss of X-Gal staining correlates to the loss of retinal ganglion cells [27]. A majority of DBA/2J mice older than 10.5 months exhibit reduced staining, relative to younger animals (closed circles). Although these mice develop progressively more damage as they age, there is a high degree of variability in the amount of damage exhibited by mice at the older ages. Retinal disease is associated with the DBA/2J genetic background, since no cell loss was observed in C57BL/6 mice (open circles) at any age examined.

A second cohort of 289 eyes from 145 D2 mice, heterozygous for the βGeo reporter gene (*Fem1c*^*R*3/+^), were aged and euthanized between the ages of 2.3 and 19.5 months. The retinas were stained with X-Gal and whole mounted for scoring using a semi-quantitative method. Figure 5 shows 4 retinas from different mice all aged to 13 months. Each retina representatives an example of increasing score from ~1 (Fig. 5A) to ~4 (Fig. 5D). These data also demonstrated the wide range of ganglion cell loss apparent in mice of the same age. A scatter plot of the scores for all the individual eyes is shown in Figure 6. Individual D2^R3/+ ^mice within most age groups exhibited variable staining, but in general a minority of mice aged 8–10 months exhibited signs of degeneration (41%), while the majority of mice aged 10.5 months and older (>75%) showed moderate to severe damage. As a control group, we also scored 34 eyes from 17 aged C57BL/6^R3/+ ^mice. These mice showed no loss of staining in either eye even at 16.5 months of age.

**Figure 7:**
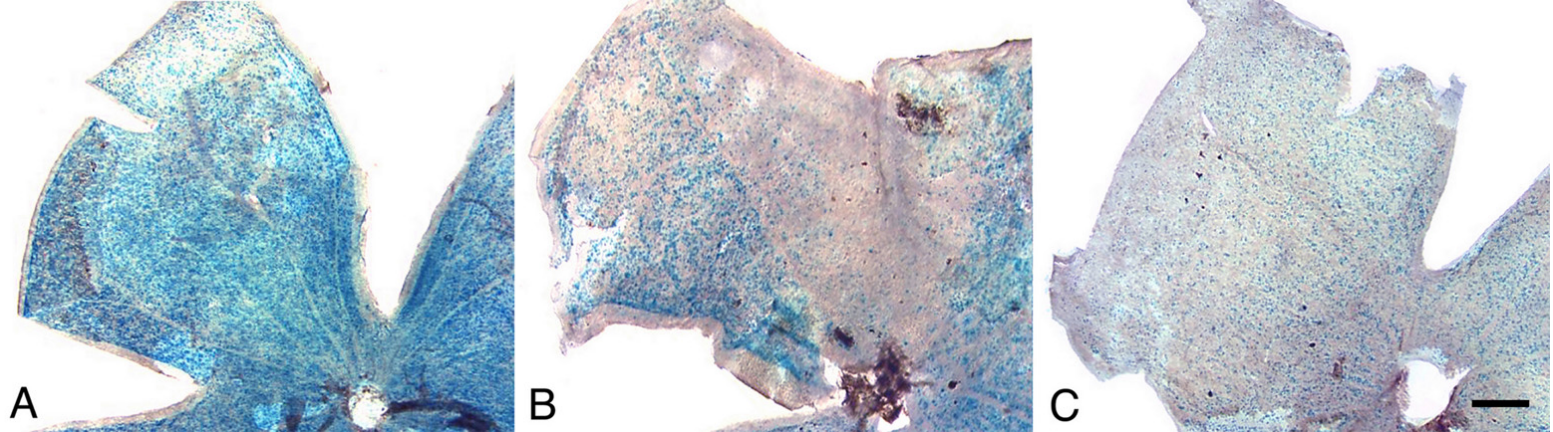
**βGeo staining pattern of DBA/2J^R3/+ ^mice showing distinct patterns of ganglion cell loss**. (A) A retinal lobe of a young mouse showing normal staining for βGeo activity. (B) A retinal lobe from an older mouse (9 months), showing regional loss of ganglion cells in a sector of retina with adjacent regions of normal retina. (C) A retinal lobe from an old mouse (11.5 months) showing diffuse loss of ganglion cells generally uniformly across the retina. In this cohort of mice, the pattern of cell loss seen in the example in (B) was exhibited principally in middle-aged mice (8–9 months), with early signs of degeneration. Bar = 0.5 mm.

Using the R3 marker also allowed us to examine the pattern of cell loss over the whole retina. Essentially two patterns of loss were observed (Fig. 7). Mice aged between 8.5 and 10 months often showed regional cell loss, typically in wedge-shaped patterns extending from the optic nerve to peripheral retina. Older mice often exhibited more uniform cell loss, suggesting that areas of regional loss may coalesce as the disease progresses. For statistical analysis, we selected all the retinas that were scored as 1.5 or greater as being eyes with at least some degree of damage. Scores for individual lobes were compared to determine if particular regions of the retina were more susceptible than others to degeneration. No significant association was found (*P *= 0.75, ANOVA) indicating that regional loss occurred randomly around the retina. We then compared scores for peripheral and central retinal regions. In this analysis, we also found that cells were not preferentially lost in either retinal region (*P *values ranged from 0.72 to 0.99 by ANOVA, when individual regions of the retinas were tested separately).

**Figure 8:**
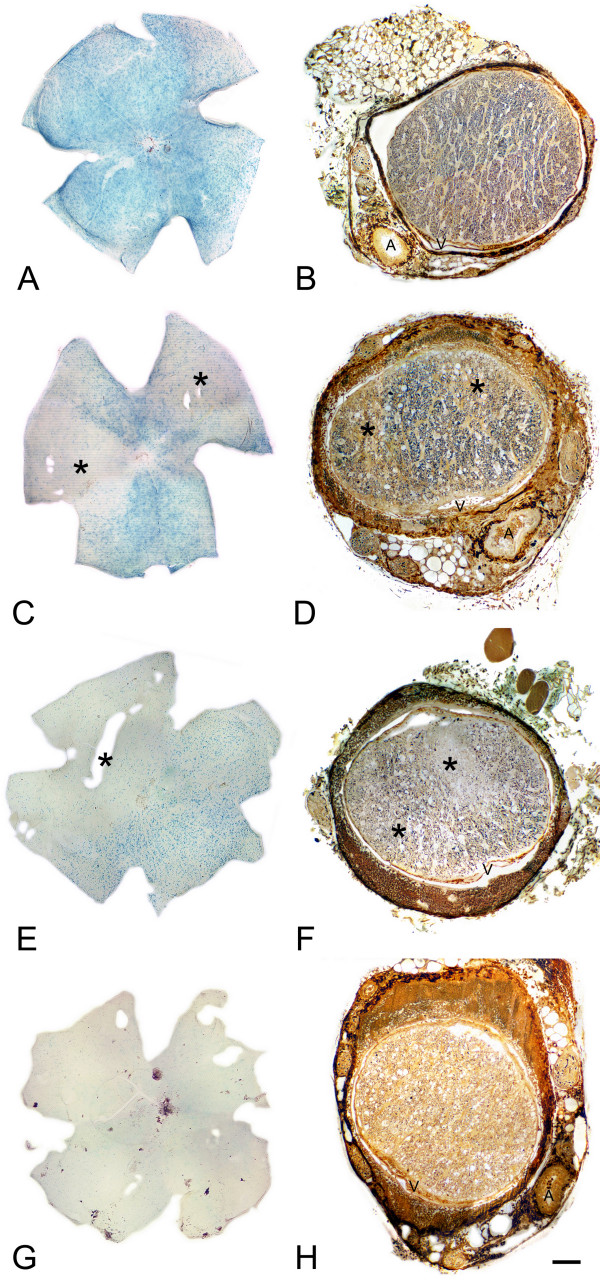
**Retinas and optic nerves show consistent patterns of degeneration**. A series of retinas and corresponding optic nerves from 4 different mouse eyes. The retinas were stained for βGEO enzyme activity and the optic nerves were sectioned just posterior to the laminar region and silver-stained. (A, B) A retina with extensive staining has a nerve with normal appearing nerve with well-defined bundles of axons. (C, D) A retina with two wedge-shaped regions of cell loss (asterisks) has a nerve with two similar focal areas of axon loss (asterisks). (E, F) A retina with a large region of cell loss (asterisk) in one half and uniform cell loss in the other has a nerve with a large contiguous region devoid of axons (asterisks), while the remainder of the nerve has a uniform depletion of axons. (G, H) A retina with nearly complete cell loss has a nerve with nearly complete axon loss. Each retina is oriented with the superior region to the top. Each optic nerve is oriented with the dorsal nerve to the top. The central retinal artery (A) and vein (V) are indicated. Bar = 0.45 mm (panels A, C, E, G) and 90 μm (panels B, D, F, H).

The pattern of axon loss in the optic nerves of mice exhibiting different patterns of retinal degeneration was also examined (Fig. 8). Mice with wedge-shaped regions of loss in their retinas exhibited similar regions of focal axon loss near the laminar region of the optic nerve, where axon tracts were still in discrete bundles. The focal nature of these regions was dissipated in sections of the proximal segments of the same nerves (data not shown). A pattern of diffuse cell loss was associated with a similar pattern of diffuse axonal loss in the optic nerve.

### The asymmetry and timing of degeneration in D2 mice

**Figure 9:**
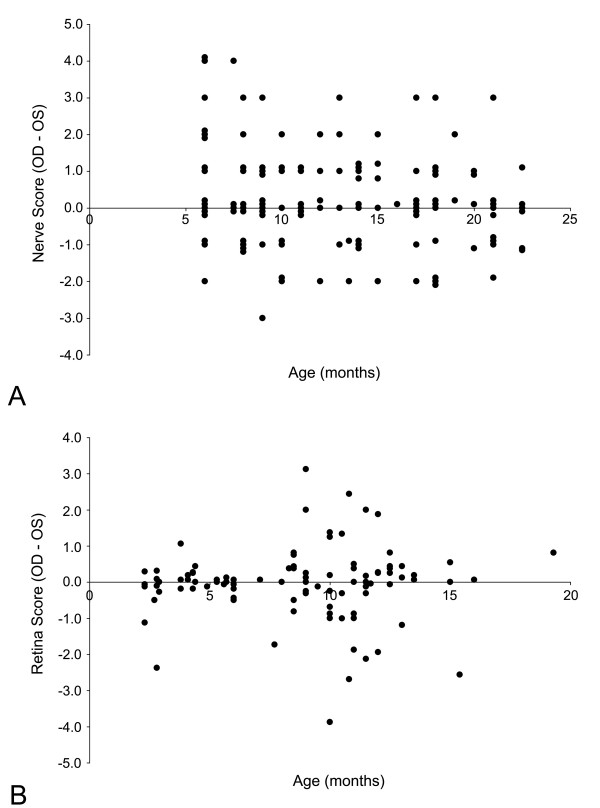
**Optic nerve and retina degeneration in DBA/2J mice is asymmetric**. Scatter plots showing the difference in both optic nerve scores (A) or retina scores (B) for individual mice. The difference in score was calculated by subtracting the left eye (OS) score from the right eye (OD) score for each mouse. The expected result for symmetric degeneration would be a score of '0' for each animal, but clearly there is dramatic scatter of both the optic nerve and retina scores. Optic nerves also show a trend for asymmetry at an early age, consistent with the hypothesis that early damage occurs first in the nerve. Retina asymmetry is more pronounced at ages when the mice show clear signs of retinal degeneration.

During the evaluation of the optic nerves and retinas from individual mice, it was evident that some animals showed dramatic asymmetry in both optic nerve disease (see Fig. 1) and loss of ganglion cells in the retina. To assess the extent of asymmetry in optic nerve degeneration, the score for the left optic nerve was subtracted from the score for the right nerve and graphed as a scatter plot (Fig. 9A). Nerves that differed by less than 1 were considered symmetric, since this was generally the maximum difference in retinal scores given by masked observers for samples that they disagreed upon. Our analysis included mice with nearly end-stage degeneration of both nerves, thus we likely underestimated the asymmetry of disease because there was no way to assess how rapidly each individual nerve had degenerated in these animals. Of the 135 mice in this cohort, 81 (60%) exhibited asymmetric optic nerve degeneration, with no significant preference between the left and right eyes (*P *> 0.10, χ^2 ^analysis). A total of 11 mice (8.1% of the entire cohort) exhibited complete asymmetry with one degenerated nerve and one normal nerve.

The difference in retinal score between eyes of the same mouse was also calculated and graphed (Fig. 9B). Eyes that differed by less than 0.5 on our grading scale were considered the same based on the level of variability observed in scorer agreement analysis. Forty percent of the mice (58/145 mice) had scores that differed between the eyes by more than 0.5 on our grading scale, again with no significant preference between left and right eyes (*P *> 0.50, χ^2 ^analysis). A total of 13 mice (9% of the entire cohort) had scores that differed by more than 2.0 on the grading scale, which was similar to the percentage of mice exhibiting complete asymmetry of optic nerve degeneration.

## Discussion

The results from two separate cohorts of aging D2 mice are reported here. In the first cohort, anterograde DiI-labeling and confirmatory histology showed a marked increase in degeneration of the optic nerve in a majority of mice when they reach 9 months of age. In the second cohort, ganglion cell loss was followed as a function of histochemical staining activity of the ROSA3 (*Fem1c*^*R*3/+^) βGeo reporter gene product. Using this method, a majority of mice exhibit moderate to severe damage by 10.5 months of age.

### Degeneration of the optic nerve

The timing of optic nerve degeneration we observed in our mice followed a similar pattern recently reported by Libby et al. [5], with the exception that we detected signs of degeneration at 9 months of age, or 1 month earlier than the Libby study. A variety of factors may account for this difference, including different sensitivities in the methods used to evaluate the nerves, the numbers of mice analyzed at critical ages, and different housing and feeding conditions that could affect the timing of the disease in different colonies.

By combining the data collected using silver staining of optic nerve sections and the DiI-labeling technique, we speculate that axons damaged in glaucoma start to degenerate in regions throughout the nerve, proximal to the laminar region. The overall pattern of degeneration, however, occurs in a retrograde direction. A similar pattern of retrograde degeneration has been reported in tibial nerves of mice after nerve crush [13]. Interestingly, the pattern of degeneration of axons is different if a more acute lesion (transection) is performed on the tibial nerve. In this latter paradigm, Wallerian degeneration occurs in an anterograde direction and is initiated more rapidly than in nerves after crush. The direction of degeneration we observe in D2 mice suggests that the nerve lesion in glaucoma is more akin to crush in severity.

In addition to the retrograde direction of loss, we also observed the "preservation" of peripheral tracts of axons in a significant number of nerves examined. The lack of uniform degeneration across the whole diameter of the nerve suggests that damage to the axons is probably more focal than diffuse. A similar condition has been reported in the nerves of humans with glaucoma [14-17] and non-human primates with experimental glaucoma [18]. In these cases, glaucomatous degeneration followed a classic "hourglass" pattern of axon loss in the inferior and superior regions of the optic nerves, which has been associated with structural differences in the collagen beams of the lamina cribrosa [15]. Similarly, mice with experimental ocular hypertension (as apposed to the chronic disease exhibited by D2 mice) exhibit initial axon loss in the superior optic nerve [19]. Although we have not detailed any predictable pattern of loss in DBA/2J mice, when present, the preserved axon tracts are found predominantly in the nasal optic nerve. Structural studies of the optic nerve head in some strains of mice have not found any evidence of an extracellular lamina cribrosa [20], but this does not preclude the possibility that mice also have some kind of increased structural support in different regions of the optic nerve.

The method of DiI-labeling also allowed us to rapidly evaluate the symmetry of optic nerve damage in each mouse. A majority of animals exhibited more damage in one nerve over the other, with no preference between left and right eyes. Like the non-uniform degeneration of axons we observed in some optic nerves, asymmetric degeneration between eyes is also similar to the natural history of this disease in humans. Several independent studies in which visual field defects and changes to the optic disc were analyzed, have reported marked asymmetry of disease progression in glaucoma patients [21-25].

### Degeneration of the retina

Using βGEO activity to mark ganglion cells, the earliest detectable changes in the retina we observed were at 8–10 months of age, with a peak in the number of animals showing degeneration occurring by 10.5–11 months of age. Our data showing peak disease at this age are consistent with the TUNEL data reported by Libby et al [6] and the loss of fluorogold-labeled ganglion cells in DBA/2NNia mice, a substrain of DBA/2J [8], indicating that the progression of disease is not unduly influenced by the presence of a single R3 allele. Our results contradict the observations made by Scheuttauf and colleagues [12], however, in that we found no obvious increase in cell loss in 6 month old mice. Since the Scheuttauf group did not quantify the amount of cell loss they observed, it is not known if the reported increase in cell death they observed was large enough to detect using the βGeo staining method. It is also clear that the timing of retinal degeneration is highly variable in a population of aging D2 mice and because this other study used relatively small numbers of mice for their studies, their data may not be representative of the general population.

The βGeo staining method also provided us with some insight on the pattern of cell loss exhibited in the D2 retina. The majority of mice showed a diffuse pattern of loss throughout the retina. Many mice in the early stages of retinal degeneration, however, clearly showed regional cell loss, often in the form of large pie-shaped sectors of retina extending from the optic nerve to the periphery. An identical pattern of ganglion cell loss was independently observed in aged D2 mice by Jakobs and colleagues [10] and may be consistent with the "patchy" cell loss reported in DBA/2NNia mice by others [8,9], although these mice appear to lose cells preferentially from the central retina rather than in sectors [9]. In each of these studies, ganglion cell depleted regions of the retina were typically bordered by normal regions of retina, suggesting that damage had occurred initially to discrete bundles of axons in the optic nerves of these animals. Since the only region of the nerve where discrete bundles exist is in the laminar region (see Fig. 3), this is the likely site of initial insult in glaucoma. Histological evaluation of the laminar regions of nerves from eyes with regional cell loss also showed similar patterns of discrete areas of axon loss supporting this hypothesis.

Like the optic nerve studies, we also observed asymmetry in the loss of βGeo positive cells in the two eyes of single mice, indicating that the disease progresses independently in each eye. This finding is also consistent with ERG response changes reported in the DBA/2NNia mice [7], where the decrease in amplitude of the b-wave form was much more variable between the two eyes of aged (15 month) mice compared to younger animals. Although the exact cause of the ERG changes was not elucidated, the authors attributed it primarily to retinal damage.

### Comparison of the timing of optic nerve and retinal degeneration

**Figure 10:**
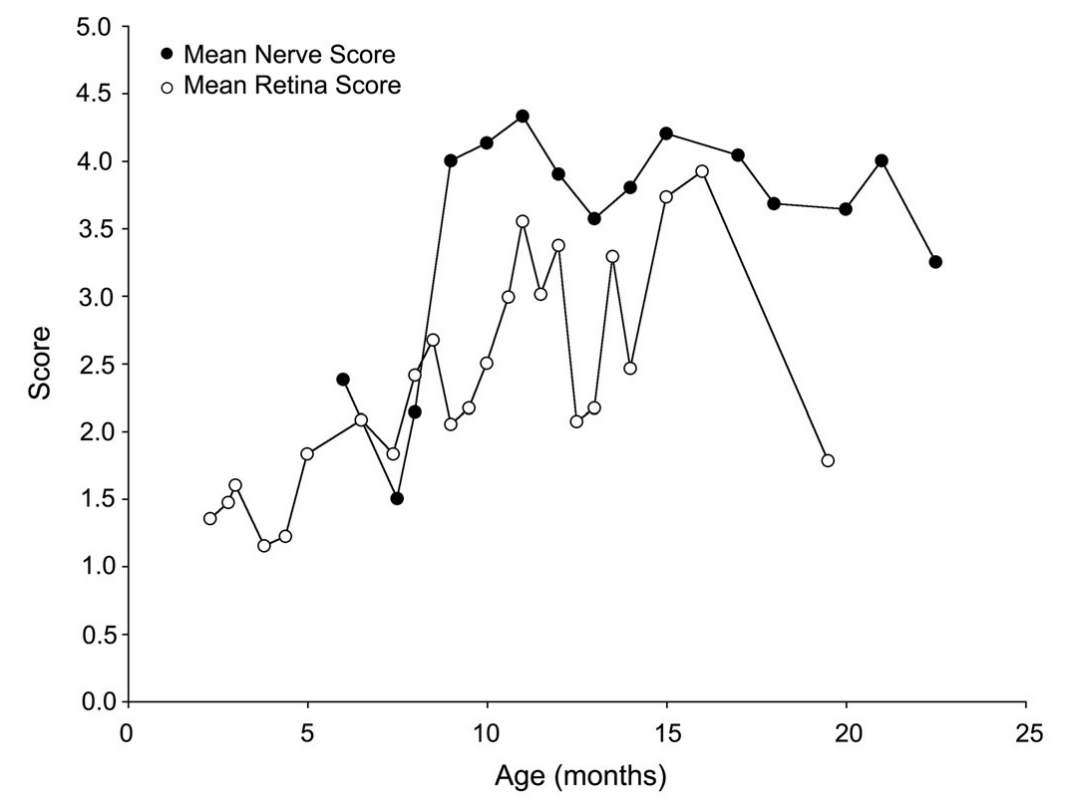
**Peak optic nerve damage precedes peak retinal damage in DBA/2J mice**. Line graph of the mean optic nerve degeneration (closed circles) and mean retinal degeneration (open circles) as a function of age in two cohorts of DBA/2J mice. In this comparative analysis, DBA/2J mice exhibited optic nerve disease before they exhibited retinal disease, consistent with theories of human glaucoma that predict that the initial damage in response to ocular hypertension occurs at the level of the lamina cribrosa [14, 17, 30].

We compared the time course of both optic nerve and retinal degeneration in an effort to estimate if one preceded the other. Figure 10 shows a plot of the mean optic nerve scores and the mean retina scores plotted relative to age. Signs of optic nerve disease were observed 1–2 months before detectable ganglion cell loss in the retina. This comparative analysis suggests that the initial damage to ganglion cells in this model occurs to their axons in the optic nerve. One caveat with this comparison, however, is that we used two independent cohorts of mice to assess optic nerve and retinal disease. Secondly, since we used ranks based on non-linear criteria for determining the onset of degeneration, we cannot be certain that early onset retinal degeneration may be found to occur at a younger age when examined using a more linear method of quantification. Our data are consistent, however, with recent observations that ganglion cells with relatively normal somas exhibit shrinkage of their axons and fail to label by retrograde transport of vital dyes injected into the superior colliculus, also suggestive of early axonal damage [10].

## Conclusion

In summary, this study of the pattern of optic nerve and retinal degeneration in D2 mice provides some interesting insights into the sequence of events in the pathology of the disease. Analysis of the timing of both optic nerve and retinal degeneration suggests that the nerve is affected first. Axons begin to degenerate posterior to the lamina in intermittent regions throughout the nerve, but are more prevalent in the proximal regions, making the overall pattern of degeneration appear retrograde by DiI staining. The loss of ganglion cells in the retina is delayed by about 1 month, and often first occurs in discrete wedges of cells. Since damage likely occurs first in the optic nerve, it is reasonable to assume that the discrete pattern of damage observed in the retina is due to damage to adjacent bundles of axons, which are only found in the laminar region. Thus, the lamina is probably the initial site of damage in glaucoma in these mice, analogous to previous studies suggesting a similar etiology in humans [14,15,17].

## Methods

### Handling of animals and generation of DBA/2J ROSA3 mice

Experiments using mice were conducted in accordance with the Association for Research in Vision and Ophthalmology statement on the use of animals for ophthalmic research and the University of Wisconsin Animal Care Committee. A colony of D2 mice was established from breeders purchased from the Jackson Laboratory (JAX – Bar Harbor, ME) and routinely backcrossed onto new founders from JAX in order to reduce genetic drift in the colony. The ROSA3 line of mice was initially generated in the laboratory of Philip Soriano [26] using the promoter trap gene βGeo (a fusion protein of β-galactosidase and neomycin phosphotransferase). In this line, the βGeo trap gene was inserted into the first intron of *Fem1c*. Adult animals express this gene in distinct populations of neurons in the CNS. In the retina, βGeo is expressed predominantly in the majority of retinal ganglion cells [27]. The ROSA3 allele (*Fem1c*^*R*3 ^- R3) was crossed into the D2 background by successive breeding through 10 generations. D2 mice, heterozygous for the R3 allele, were generated by crossing homozygous animals (*Fem1c*^*R*3/*R*3^) with wild-type D2 mates obtained from JAX. Mice were maintained on a 4% fat diet (8604 M/R Diet, Harland Teklad, Madison, WI) in a 12 hr light/dark cycle.

### Post-mortem DiI labeling of optic nerves

**Figure 11:**
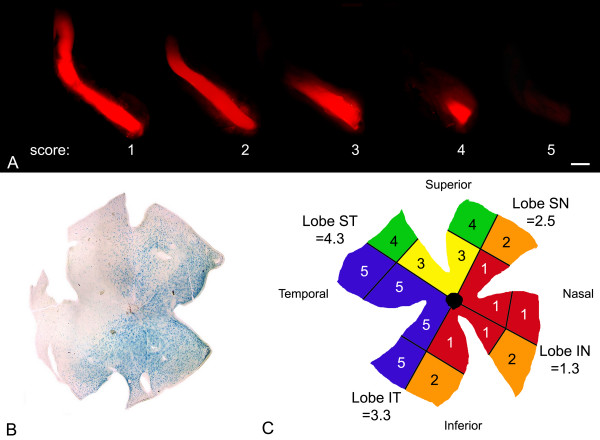
**Summary of the scoring criteria for DiI-labeled nerves and X-gal stained retinas**. (A) Exemplar of scored optic nerves. Only the left nerve is shown for 5 individual mice. The scores range from 1 for label from the globe to the chiasm, to 5 for no signs of label. Younger mice typically exhibited nerves that were scored 1–2, while older mice typically had nerves showing some level of degeneration (3–5). Bar = 0.5 mm. (B) A photomicrograph of a retina (OD) stained for βGEO activity taken from a 10.5 month old mouse. This particular example appears to have 2 wedges of cell loss, one superior and one temporal, at different stages of degeneration. (C) A cartoon of a flatmounted retina where each quadrant is separated into 4 regions and given a score based on the intensity of stain present. The quadrants represented are (clockwise): SN, superonasal; IN, inferonasal; IT, inferotemporal; ST, superotemporal. The scores in each region of the quadrants represent the scores given by 1 masked observer for the retina in (B). The average of all the scores in each quandrant then becomes the total score for that quadrant, and the average of these 4 scores becomes the final total score for that retina.

Axons in the optic nerves of D2 mice were labeled post-mortem with DiI crystals (Molecular Probes, Eugene, OR) using a modification of the procedure described by Plump et al [28]. Briefly, adult mice between 6 and 21 months of age were euthanized. The heads were removed and fixed in 4% paraformaldehyde in Phosphate Buffered Saline (PBS) for 1 hr at room temperature. After fixation, the heads were skinned and a 270° incision was made around the circumference of the globe at the limbus of each eye to allow the corneas to flip open. The lenses were removed, and crystals of DiI were embedded into the optic nerve head of each eye using watchmaker's forceps. To keep the crystals in place, the lenses were replaced and the corneas folded back into position. Heads were incubated for 2 weeks in PBS containing 0.1% sodium azide at 37°C to allow the DiI to diffuse along the axon plasma membranes. After incubation, the skullcap and underlying brain tissue was removed to expose the optic nerves from the globe to the chiasm. Fluorescent label in the nerves was visualized using a Leica MZ FL III fluorescent dissecting microscope with a digital camera attachment (Leica, Bannockburn, IL). To estimate the staining intensity of each nerve, individual nerves were scored by 2 masked observers using a 5 point scale ranging from label extending from the globe to the optic chiasm (score of 1) to no detectable label in the entire nerve (score of 5). An exemplar of the 5 different scores is shown in Figure 11. A weighted Kappa statistic showed a high level of agreement between the 2 observers (κ = 0.818, 95% CI = 0.697 to 0.939).

### Optic nerve histology

After DiI staining and imaging, optic nerves were removed from the mouse heads and processed for histology. Nerves were dissected with a small region of the globe still attached to help define regions proximal and distal to the retina. Nerves were fixed for 4 hr at 22°C in 10% Formalin in PBS, embedded in JB-4 Plus plastic (glycol methacrylate, Polyscience, Warrington, PA), and sectioned at 2 μm thickness. Cross sections were taken within 1.0 mm of the globe (distal segment) and within 1.0 mm of the chiasm (proximal segment). Some nerves were also sectioned longitudinally. Sectioned nerves were stained using a silver impregnation technique, which selectively stains the axons [29]. Optic nerves were digitally photographed using an Olympus BH-2 photomicroscope (Mellville, NY).

### Histochemical staining for βGeo enzyme activity and scoring of retinal wholemounts

Mice were euthanized at the appropriate ages and the superior region of each eye was marked using an ophthalmic cautery to place a small burn in the cornea. Eyes were then enucleated, and the anterior chamber and lens of each was removed after a relaxing cut was made in the superior retina in line with the cautery mark. Retinas were stained with X-Gal (1 mg/mL) as described previously [27], with the exception that enucleated eye cups were stained for a standard period of 21–22 hrs at 37°C in an effort to ensure all eyes were stained an equal period of time. After staining, retinas were removed from the eye cups and flat mounted on charged glass Plus microscope slides (Fisher Scientific, Chicago, IL) using care to maintain retinal orientation based on the superior retina. Three additional relaxing cuts were then made to flatten the tissue onto the slide. This procedure resulted in retinas with 4 relatively equal sized lobes corresponding to the superonasal, superotemporal, inferonasal, and inferotemporal quadrants. To estimate the staining intensity of each retina, each individual lobe was scored separately by 3 observers using a 1 to 5 point scale. Briefly, each lobe was divided into 4 regions (2 central and 2 peripheral) and each region was scored independently from 1 to 5. A score of 1 was given to retinas with a dense staining pattern. A score of 2 was given to retinas that exhibited reduced, but still widespread distribution of positive cells. Correlative cell counts from sample retinas indicated that these retinas had at least 50% of the positive cells in the ganglion cell layer remaining. A score of 3 was given to retinas with markedly reduced staining, to a maximum of approximately 75% cell loss. A score of 4 indicated sparse distribution of positive cells in the ganglion cell layer, while a score of 5 was given to retinas with no evidence of positive cells in the ganglion cell layer. The mean of the 4 scores for each lobe was considered the score for that lobe, and the mean score of each of the 4 lobes was considered the mean score for that retina. This scoring method created data sets that enabled statistical analysis of cell loss between the peripheral and central retina, between different lobes of each retina, and between different retinas. An example of a scored retina is shown in Figure 11. A weighted Kappa statistic revealed a high level of agreement among the scorers using this method (κ = 0.667, 95% CI = 0.32 to 1.0).

## Authors' contributions

CLS and RWN (corresponding author) jointly conceived and designed this study, analyzed all the data, and wrote the manuscript. CLS and JAD developed the retina scoring system and KTJ contributed to the scoring and statistical evaluation of the retina cohort. RWN and YL developed the optic nerve scoring system. YL did the DiI staining experiments and optic nerve histology. YL and JAD did the retina staining experiments. All authors read and approved the final manuscript.
